# Research and Simulation on the Development of a Hydraulic Prop Support System of Powered Roof Support to Increase Work Safety

**DOI:** 10.3390/mps7020033

**Published:** 2024-04-11

**Authors:** Beata Borska, Dawid Szurgacz

**Affiliations:** 1 KWK Ruda Ruch Halemba, ul. Halembska 160, 41-717 Ruda Śląska, Poland; borskab@gmail.com; 2 Center of Hydraulics DOH Ltd., ul. Konstytucji 147, 41-906 Bytom, Poland; 3 Polska Grupa Górnicza S.A., ul. Powstańców 30, 40-039 Katowice, Poland

**Keywords:** extreme mining environmental monitoring, optical sensors, simulations, bench tests, tests under real conditions

## Abstract

The underground mining environment is currently based on technology that uses mainly analogue sensors in machine and equipment control systems. The primary machine performing the most important functions in a mining system is the powered roof support. In order for it to work properly, it is important that it achieves the required power. To ensure this, it is necessary to continuously and precisely monitor the pressure in the under-piston space of the prop. Due to the extreme environmental conditions, pressure sensors should have high sensitivity, large transmission capacity, small size and light weight. To achieve these requirements, the authors of the article propose to implement a monitoring system based on photonics technology. To achieve this goal, several studies were carried out. The range of these studies included simulations, bench tests and tests under real conditions. The obtained test results showed the possibility of developing the control system for the powered roof support, the additional function to supercharge power. Based on the analysis of the obtained test results, assumptions were developed for the development of a power charging system with monitoring sensors. Based on the guidelines obtained from the research results, thedevelopment of the above prototype based on photonics technology is proposed.

## 1. Introduction

Mining is a demanding industry, and its challenges are becoming greater every day. The economy of production and ensuring safety are essential for the success of mining activities [1,2]. In addition, an increasing emphasis is placed on the environmental aspect, and mining companies are expected to reduce their negative environmental impact [3]. Therefore, the extraction of raw materials requires constant development and adaptation to new conditions and legal regulations. Efficient and effective operation is necessary to ensure economic viability [4]. Therefore, efforts are being made to reduce machine failures, to improve machine diagnostics [5,6,7] and to assess quality [8,9]. When it comes to safety, new technical solutions are being sought to reduce risks from machinery [10], and efforts are made to combat natural hazards more effectively [11,12,13]. In addition, the mining process is being automated to improve safety and productivity [14,15,16]. In turn, in the environmental aspect, activities are aimed at reducing energy consumption [17] and encouraging the better use of by-products [18]. The latest tools and technologies are used for this aim, such as RFID technology, laser scanning, single-wire energy transfer, wire strain gauges, ultrasonic systems and preliminary estimation [19,20,21]. In addition to bench, experimental [22] and real-conditions testing [23,24], simulation studies and numerical analyses [25,26,27,28] play an increasingly important role. Statistical analyses, data analyses [29] and decision-making procedures are used. Significant development is observed in copper ore mining. The search for and implementation of innovative solutions is also observed in hard coal mining, especially in the area of the longwall mining process [30].

**Figure 1:**
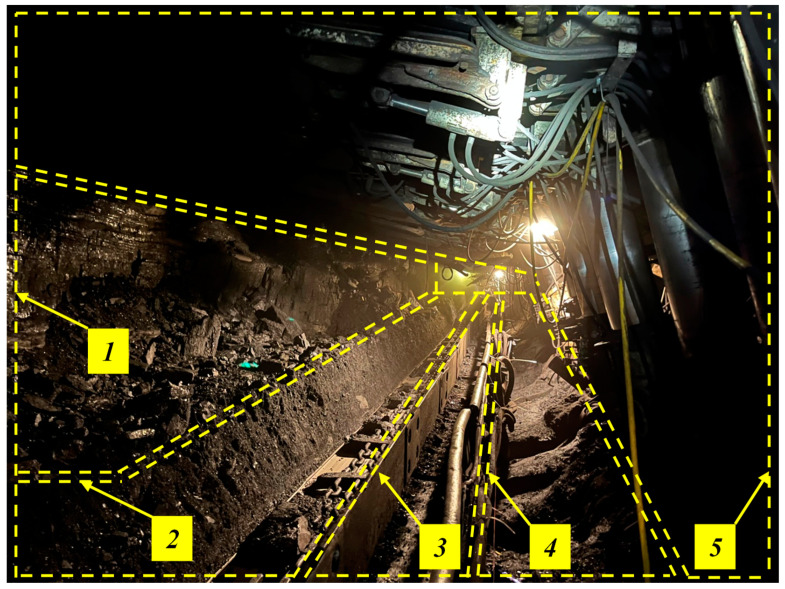
View of a mining wall using a mechanized longwall complex, where its significant areas are marked: (1) coal sidewall, (2) path of the combine shearer, (3) longwall scraper conveyor, (4) crew passage path and (5) powered roof support.

In coal mining, the most significant emphasis is placed on developing powered roof support complexes, which are most often the essential equipment of the mining wall [31,32]. The wall complex consists of a mining machine (coal miner or planer), a wall conveyor (removal of coal from the wall) and a powered roof support (see Figure 1). The second important element is the conveyor belt chain [33,34]. Belt conveyors remove the mined coal from the wall pavement under the mining shaft. Failure of one of these machines causes a halt in operation and a pause in the extraction process. Therefore, to maintain the continuity of operation, all of these machines must work without failure. At the same time, decreasing deposits and the need to reach for increasingly difficult coal deposits generates new problems and requirements for the machines and technologies used [35,36,37]. In the research area of coal extraction, the problem of adapting current technologies and machines to new mining and geological conditions is researched worldwide [4,38,39,40].

The authors of this paper focused on the development of powered roof support. The powered roof support protects against the fall of the roof rocks, providing security in the operated wall [41,42]. In addition, it assures the movement of the entire complex along the progress of the longwall’s face. Thus, it constitutes footing for the other two machines of the complex. The crew’s safety and maintenance of the operational continuity depend on the reliability of the powered roof support. The powered roof support also has the highest financial value in the mechanized complex. Due to its function and its value, the powered roof support is a crucial element of the complex. The optimal choice of powered roof support and its proper operation are essential factors for the efficiency of the entire complex.

**Figure 2:**
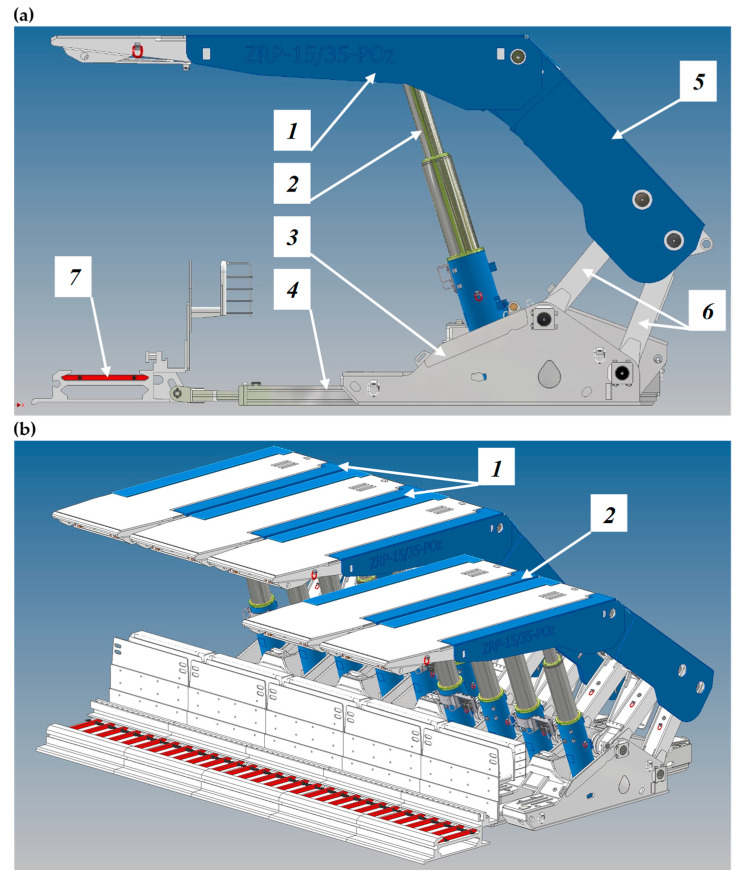
View of the powered roof support in the mining longwall: powered roof support components (**a**), canopy (1), hydraulic actuator (2), floor base (3), beam of sliding system (4), shield support (5), lemniscate mechanism (6) and longwall scraper conveyor (7); and operation of powered roof support (**b**), sections of powered roof support expensed in the wall (1) and sections of the powered roof support moved (2).

The powered roof support installed in a wall excavation consists of several dozen to more than one hundred and fifty sections (see Figure 2). The powered roof support section is made of a steel structure controlled by hydraulic cylinders. During exploitation, the sections perform repetitive operation cycles. Each cycle consists of the following steps: drawing off, moving, expanding the powered roof support and securing the excavation. Drawing off consists of decreasing the height of the section so that the cap piece no longer touches the excavation’s roof. It allows for the sections to be moved along the progress of the longwall. For longwalls using a coal miner, the section moves by a section equal to the web depth of the coal miner. After displacement, the section is expanded between the footwall and the excavation roof so that the cap piece can touch the roof again. Thanks to this, the powered roof support can again take over the pressure of the roof rocks, securing the excavation. The hydraulic props ensure the drawing off and expansion of the powered roof support. In turn, the displacement system with a cylinder is responsible for moving the sections. The props and actuators are controlled manually or in an automatic system depending on the type of control used.

The powered roof support’s continuous development and systematic changes are necessary [43,44,45] to ensure its correct operation. Development is needed due to increasingly difficult mining and geological conditions. The operation of increasingly lower decks generates greater loads on the part of the rock mass [46,47]. More and more longwalls are operated under the threat of rock mass tremors [48,49]. Additionally, the more intense coal exploitation requires reliable operation of the powered roof support. Therefore, it is necessary to optimize the powered roof support continuously. Changes are being systematically introduced in the field of load-carrying capacity, hydraulic solutions, control systems and monitoring systems for the work of the sections [50,51]. The authors took up the issue of optimizing the support system of a hydraulic prop. The change aims to improve the powered roof support’s reliability and maintain the required load-carrying capacity.

To achieve the above, the authors of the article propose the development and implementation of an innovative powered roof support monitoring system with a power charging function. For this purpose, a three-stage scope of research was adopted. Simulation tests will allow for determining the operating characteristics of the prototype for the implementation of the charging function. Based on the simulations performed, a prototype will be created. This prototype will be subjected to bench tests to confirm its correct operation. The final stage will be operational tests that will verify the suitability of the prototype in real conditions. At the same time, the obtained results of real tests will allow for setting guidelines for the development of photonic sensors dedicated to the work performed in underground mining excavations. Sensors, apart from their small size and low weight, should be characterized by high-sensitivity and -resolution measurements as well as resistance to harsh environments [52,53,54,55]. This will allow for the precise monitoring the parameters of the powered support and monitoring the operation of the rock mass within the mining wall.

This section, Section 1, concerns the powered roof support and its role in the coal mining process. This section presents its working pattern and draws attention to current operational problems. We highlight the need to adapt the powered roof support to increasingly difficult working conditions. Section 2 describes our research problem—the maintenance of the load-carrying capacity. There, we propose the prototype solution and describe the adopted methodology. Section 3 describes the research carried out to confirm the validity of the accepted concept. The study was divided into three stages: simulation, bench and real-conditions testing. Section 4 presents a comparative analysis of the results. Section 5 is a summary of the conclusions.

## 2. Materials and Methods

One of the problems in operating a powered roof support is obtaining the required value of the load-carrying capacity. The powered roof support’s load-carrying capacity is the force with which it acts on the roof of the excavation. For the operation of powered roof support, we distinguish three types of load-carrying capacity: initial, working and nominal. Immediately after the expansion, the sections acquire their initial load-carrying capacity. Then, after taking the pressure of the roof rocks, the powered roof support section works at the working load-carrying capacity. The nominal load-carrying capacity is the maximum value for which the powered roof support section is designed. Thus, the powered roof support’s load-carrying capacity can be described by the following dependence:

(1) $$F=\frac{\pi d^{2}}{4}\cdot p_{pt}\left(t\right),$$

where *F* is the powered roof support’s load-carrying capacity (N), *p_pt_(t)* is the pressure in the under-piston space of the prop (Pa) and *d* is the prop’s diameter (m).

The value of the pressure in the sub-piston space of the prop is the factor determining the value of load-carrying capacity, in addition to the design parameters. Thus, the initial load-carrying capacity will be primarily determined by the instantaneous pressure value in the main supply line. The human factor and the quality of the operator’s actions are also crucial for expanding the section. Moreover, the technical condition of the powered roof support props and the roof conditions in the mining wall significantly impact the working load-carrying capacity. Those variables affect the considerable variation in the load-carrying capacity of the sections along the length of the wall.

If the load-carrying capacity value is too low, it can lead to excessive subsidence of the roof rocks and stratifications. This increases roof pressure on the coal bed, which can cause coal to loosen and increase the exposed roof area. These phenomena can result in cave-ins and roof rocks collapsing, leading to operational pauses and economic losses for the mine. Thus, the problem of obtaining the required value of load-carrying capacity and ensuring uniform roof support along the length of the wall became the basis for our research.

### 2.1. Proposed Change in Hydraulic Prop Support System

**Figure 3:**
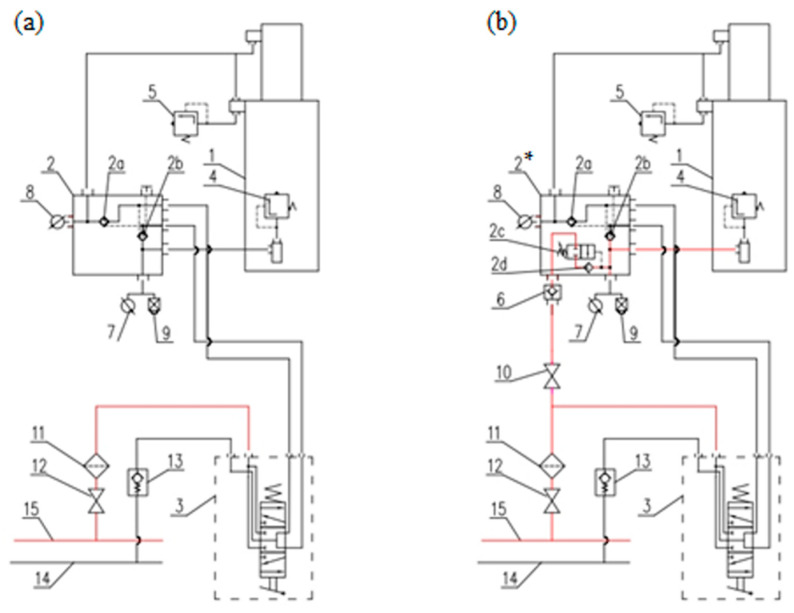
The hydraulic system of a powered roof support prop: (**a**) traditional and (**b**) modified, where 1—hydraulic prop; 2—traditional valve block; 2*—double valve block with automatic pressure charging; 2a, 2b and 2d—check valve; 2c—threshold valve; 3—four-way distributor; 4 and 5—safety valve; 7 and 8—manometer; 9—pressure indicator; 10 and 12—shut-off valve; 11—filter; 13—check valve; 14—runoff line; 15—supply line.

Changing the hydraulic system of the prop is proposed to maintain the support of the powered roof support (see Figure 3). We replaced the existing block (2) with a double block with automatic pressure charging (2*). Now, the system consists of a threshold valve (2c) with a check valve (2d) and is connected to the main supply line (15) via a second check valve (6) and a cutoff valve (10). The threshold valve (2c) installed in the block has an opening pressure setting of 9 MPa. The automatic pressure charging function does not work below this value. When the pressure exceeds 9 MPa during the expansion of the props, the charging system is switched on. The prop expansion is assured at the required initial load-carrying capacity equal to the maximum pressure value of the main supply line, despite the interruption of the operator’s expansion function.

Using a double valve block (2*) in the prop’s hydraulic system is a prerequisite for using the charging function because it prevents the pressure drop in the sub-piston space of the prop in the event of an internal leak. This is to ensure initial and working load-carrying capacity. In addition, this system is equipped with an overflow valve (5) installed in the over-piston space. This valve is designed to protect against damage to the hydraulic lines connecting the valve block to the over-piston space of the prop, constituting adequate protection against internal leaks and the resulting increase in pressure in the over-piston space to a value exceeding the resistance of the connecting wires, as well as when only one of the props is expanded. The system also uses an additional check valve (6) connected in series to this system. Using the valve eliminates the possibility of fluid backflow to the main supply line in the event of contamination of the check valve (2d).

### 2.2. Research Methodology

**Figure 4:**
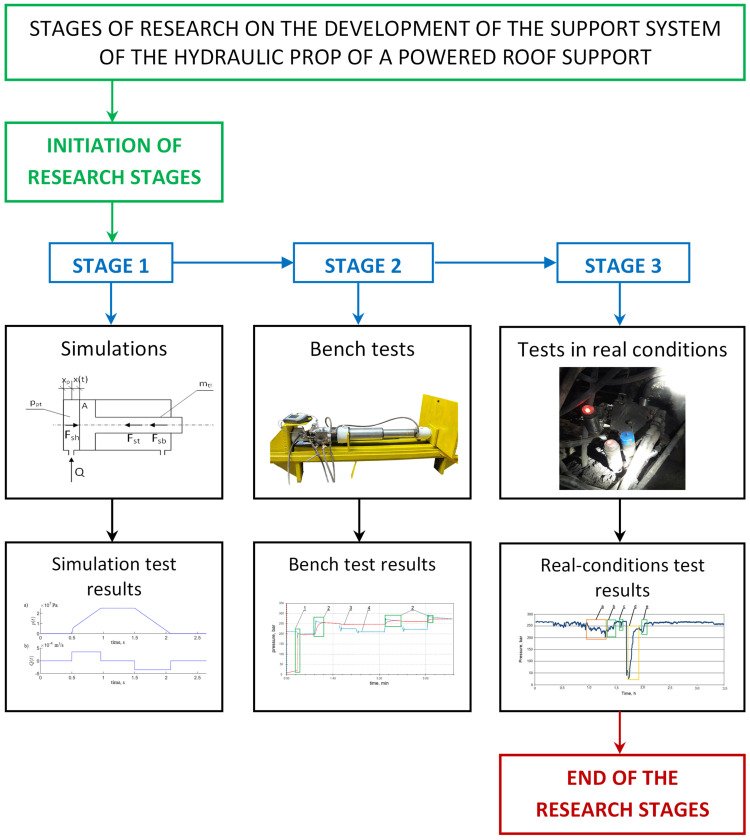
Stages of research on the development of the support system of a powered roof support hydraulic prop, where (Stage 1) (**a**)—pressure in the space under the piston of the prop, (**b**)—liquid flow rate to the prop, (stage 2) 1—expansion of the prop by the operator, 2—pressure charging, 3—pressure in the space under the piston of the prop, 4—pressure in the supply line, (Stage 3) a—pressure drop in the space under the piston of the prop, b, c, e—pressure charging, d—expansion of the prop by the operator.

Research on the prototype double block with automatic pressure charging in the powered roof support hydraulic system was divided into three stages (see Figure 4): simulation tests, bench tests and real-conditions tests. This three-step approach provides a complete assessment of the introduction of changes in the hydraulic system of the powered roof support. Simulation tests allowed us to determine the nature of the work of the future prototype. Bench tests allowed us to evaluate the correctness of the prototype’s operation. The final stage is research in real conditions to verify its usefulness.

**Figure 5:**
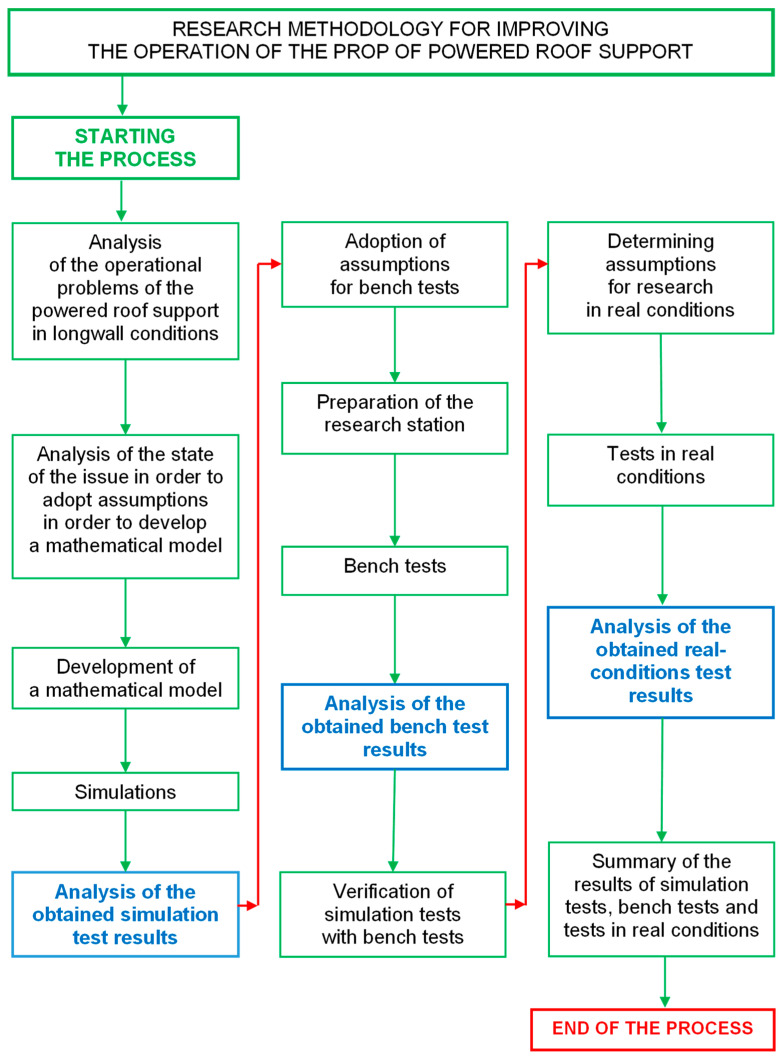
Research methodology for improving the operation of a powered roof support prop.

The accepted course of research is presented in Figure 5. The implementation of the research began with an analysis of the operational problems of the powered roof support in longwall conditions. The analysis allowed us to distinguish real problems when using the powered roof support section and the disadvantages of the currently used solutions. This analysis aimed to introduce changes in the support system of the powered roof support’s hydraulic prop. The proposed change was to provide the powered roof support with the required load-carrying capacity. The above was aimed at improving safety in hard coal mines and increasing the efficiency of the mining process.

A mathematical model was developed after analyzing the powered roof support’s operation in the longwall. The hydraulic prop’s parameters in the model largely determine the load-carrying capacity of the housing. The model, after verification, allowed us to conduct simulation studies of the work of powered roof support. Thanks to this, optimizing the currently used support system of the powered roof support prop was possible. Based on the analysis of the research results, it was possible to determine the nature of the work of the proposed prototype.

The prototype proposed in this paper was subjected to bench testing. It was necessary to develop a research plan and develop some hypotheses. Thus, a research station was prepared. The tests allowed us to verify the correctness of the prototype. They also made it possible to verify the proposed charging function. The results of bench and simulation tests allowed us to assess whether the developed solution can be the subject of further research in real conditions.

The last stage was testing under real conditions. Before starting the research, it was necessary to adopt hypotheses and determine the research plan. The research allowed us to verify the conclusions of the simulation and bench tests. Finally, the simulation, bench and real-conditions tests determined the usefulness and correctness of the proposed solution in this article.

## 3. Results

The authors conducted tests to evaluate the operation of the prototype double block with automatic pressure charging. As required by the accepted procedure (Figure 5), the scope of the study included three stages. In the first stage, simulation studies were carried out. They required the development of a mathematical model of the powered roof support. Several simulations were carried out on the model, which made it possible to verify the correctness of the adopted concept for developing a prototype block. In the second stage, the block was subjected to bench testing—the results of the bench tests allowed us to assess the correctness of the block. In the last stage, the third stage, tests were conducted under real conditions. The tests verified the work of the block in the mining wall and its usefulness.

### 3.1. Simulation Studies

**Figure 6:**
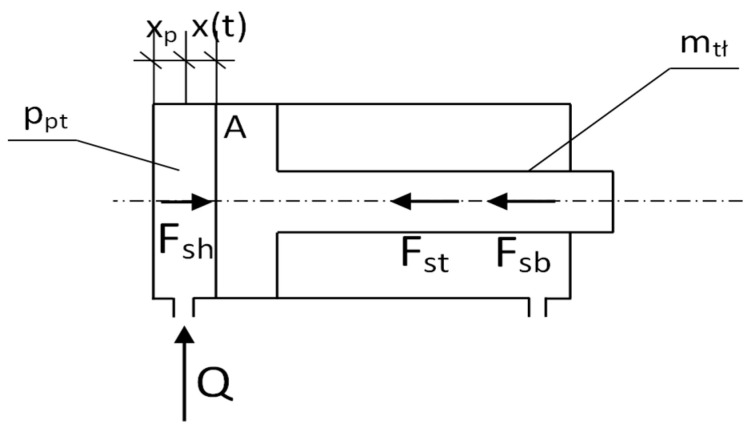
Model of a hydraulic prop for conducting simulation tests, where F_st_—the friction force; F_sb_—the force of inertia; F_sh_—the force acting on the piston; Q—the flow rate of the liquid that flows into the cylinder; A—the surface area of the piston; p_pt_—the pressure in the sub-piston space of the prop; m_tł_—the mass of the piston; x_p_—the beginning position of the piston; and x(t)—the displacement of the piston in time.

The powered roof support’s mathematical model was developed for the simulation studies. The model required the adoption of some simplifications. The mathematical model of the powered roof support was thus limited to the hydraulic prop (Figure 6). The parameters of the hydraulic prop and its technical condition have a decisive influence on the value of the load-carrying capacity of the powered roof support.

The equations for the adopted model (see Figure 6) describe the relationships in the hydraulic prop during its expansion. The mathematical model was developed based on D’Alembert’s principle and the balance of flow rates. The dependencies formed a system of Equation (2), the basis of the mathematical model:

(2) $$\left\{\begin{array}{c} -m_{tł}\cdot \frac{d^{2}x}{dt}-f_{s}\cdot \frac{dx}{dt}+p_{pt}\left(t\right)\cdot A=0\\ \frac{dp_{pt}}{dt}=\frac{B\cdot \left(Q-A\cdot \frac{dx}{dt}\right)}{A\cdot \left(x_{p}+x\left(t\right)\right)} \end{array}\right.$$

where *m_tł_*—the mass of the piston; *x*—the displacement of the piston; *f_s_*—the coefficient of friction; *p_pt_*—the pressure in the sub-piston space of the prop in time; *A*—the surface area of the piston; *Q*—the flow rate of the liquid that flows into the cylinder; *x_p_*—the beginning position of the piston; and *B*—the bulk modulus of hydraulic fluid.

**Figure 7:**
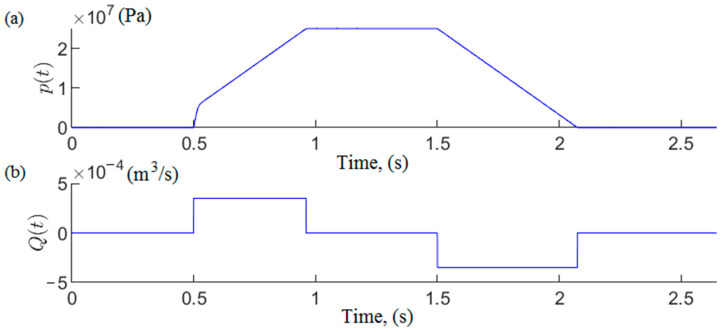
Results of simulation tests: (**a**) course of pressure changes in the space under the piston of the prop, and (**b**) the flow rate of liquid into the prop.

Simulation studies were carried out on the developed model. The MATLAB R2020b software was used for this research [56]. As a result of the computer simulations, temporal pressure changes in the prop’s sub-piston space were obtained. Sample test results are presented in Figure 7.

The model studies made it possible to obtain the characteristics of pressure changes in the sub-piston space of the hydraulic prop during its expansion. The results of the studies show that two characteristic phases can be distinguished in the expansion process. In the first phase, the actuator piston does not move. Then, the liquid inflow causes a rapid increase in pressure in the prop’s sub-piston space. When a specific pressure limit is exceeded, the force acting on the piston sets it in motion. The pressure continues to rise, but this increase is much milder.

**Figure 8:**
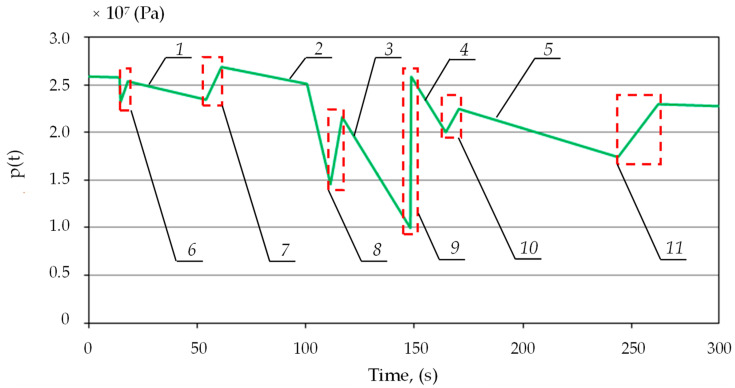
Results of simulation tests for the proposed charging function, where 1, 2, 3, 4, and 5—pressure loss; and 6, 7, 8, 9, 10, and 11—pressure charging area.

Then, according to the adopted hypotheses, the proposed pressure charging function was modeled. Based on empirical studies, the losses of liquids, in the event of a leak in the system, from the sub-piston space of the prop were estimated. A charging function was modeled to replenish the pressure in the sub-piston space to the required value. The charging function diagram is shown in Figure 8. Characterizing and modeling the charging allowed for the development of a prototype block with the charging function.

**Figure 9:**
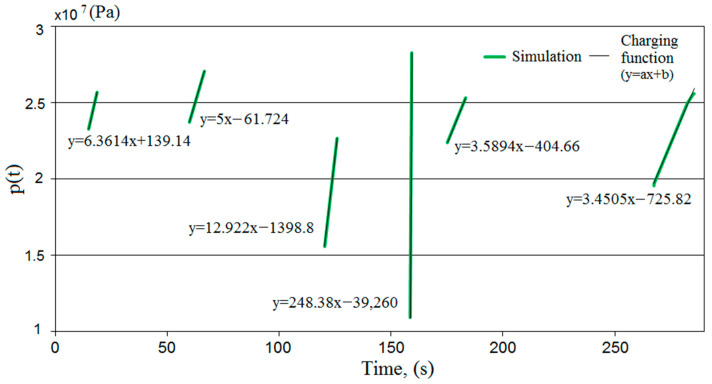
The course of the modeled charging function.

Figure 9 shows the supercharges obtained in simulation tests. These charges were described by a linear function. The slope of the function describes the charging speed. For the fourth charge, a high value of the slope coefficient was obtained, different from the others. In this case, a significant pressure drop was noted under the prop piston. Therefore, there was a significant difference in the pressure values in the sub-piston space of the prop and the supply line, which resulted in a higher liquid flow rate for charging. This could affect the speed of the charging.

### 3.2. Bench Tests

**Figure 10:**
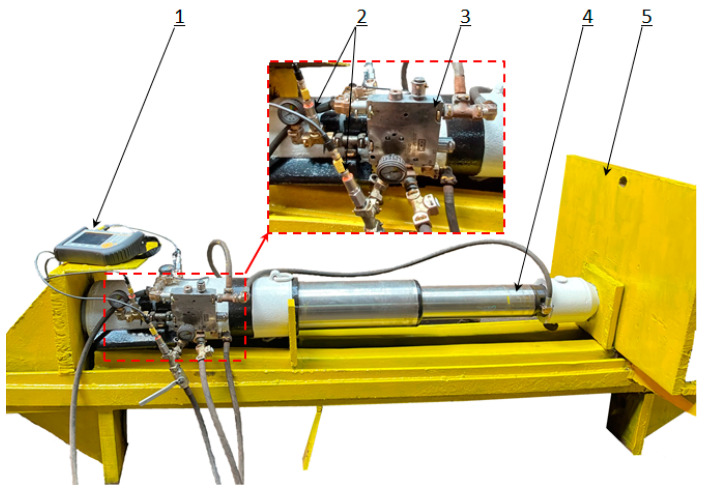
Test site with the tested double block: 1—measuring device; 2—pressure sensors; 3—double block with automatic pressure charging; 4—hydraulic prop; and 5—test site frame.

The mathematical model made it possible to conduct simulation studies of the hydraulic prop’s. Therefore, several simulations were carried out in MATLAB. The results and their analyses allowed us to determine the nature of the future prototype’s operation. On this basis, a change in the hydraulic system of the powered roof support was proposed. The change involves replacing the existing valve block with a prototype double block with automatic pressure charging. The concept and design of the double block is presented in Figure 10. Bench tests were carried out to assess the correctness of the block’s operation.

**Figure 11:**
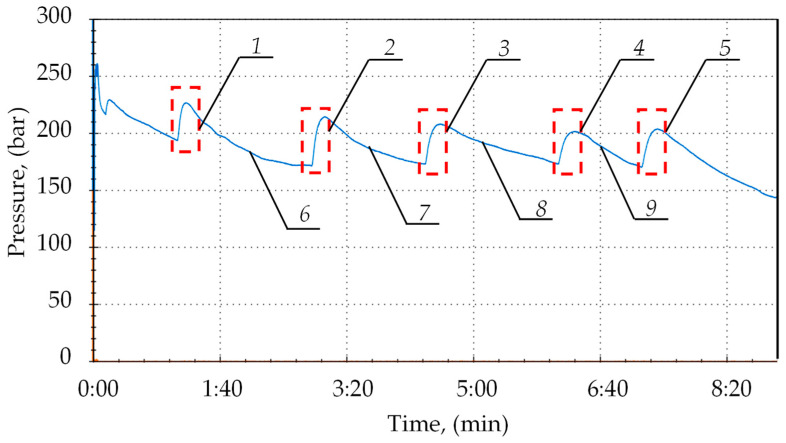
Results of bench tests of the double block operation with charging: 1, 2, 3, 4 and 5—automatic pressure charging area; and 6, 7, 8 and 9—pressure loss in the space under the prop’s piston.

Bench testing required the preparation of a test bench and a hydraulic actuator. A pump station was used to power the actuator. While testing, the pressure in the sub-piston space of the prop was measured. The pressure was measured using the Parker Service Master Plus device with a sampling rate of 0.01 ms. The first stage of bench testing verified the correctness of implementing the charging function. For this purpose, a leak was made in the prop’s hydraulic system. As a result of this leak, pressure drops occurred in the sub-piston space of the prop. An example of the pressure changes in the prop’s sub-piston space during its operation is shown in Figure 11.

During the prop’s operation, pressure drops occurred in the sub-piston space due to the external leak. These drops ranged from 5 to 15 MPa. As one can see in the chart below (see Figure 11), the block automatically started charging after each pressure drop. The automatic charging function switched on each time. Thanks to this, the hydraulic prop had no loss of load-carrying capacity despite the leaks. Using a double block with automatic pressure charging ensured that the required pressure value was maintained in the sub-piston space of the prop. Studies have shown that it is necessary to maintain the supply pressure at a minimum of 25 MPa for optimal block operation.

**Figure 12:**
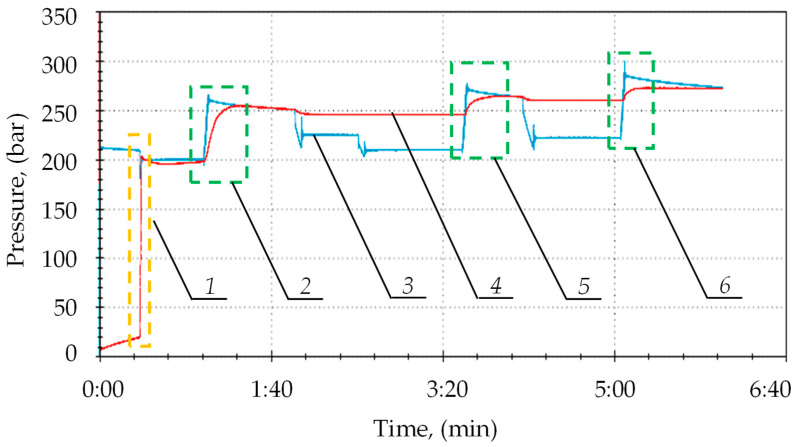
Results of bench tests of the double block operation with charging: 1—expansion of the prop by the operator; 2, 5 and 6—automatic pressure charging area; 3—pressure in the supply line; and 4—pressure in the space under the prop’s piston.

The second stage of the study allowed us to confirm the accepted thesis that the double block with automatic charging ensures that the prop is expanded to the maximum pressure value in the supply line. The test results are presented in Figure 12. During the testing, the prop was expanded by the operator to a pressure value of 20 MPa (1), equal to the instantaneous pressure value in the main supply line. The graph shows that the block automatically started charging after each increase in pressure in the main supply line (2). As a result, the pressure in the prop’s sub-piston space reached 27 MPa. It can also be observed that, with temporary pressure drops in the main supply line (3), the pressure in the sub-piston space of the prop was kept at a constant level. The positive results of bench tests allowed us to progress to the last stage, i.e., research under real conditions.

### 3.3. Tests in Real Conditions

**Figure 13:**
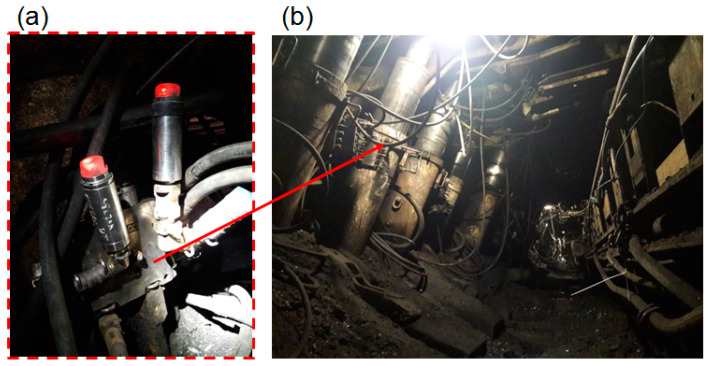
The extraction wall in which the research was carried out: (**a**) the pressure sensors with a prototype double block and (**b**) a view of the sections on which the research was carried out.

The third stage of the research allowed us to assess the usefulness of the block in real conditions (see Figure 13). At the last stage, research was carried out in a mining wall. The operation was carried out in a longitudinal wall system by the fall of the roof. The depth of operation was 780–850 m. The wall was 166–245 m long and had a run of 970 m. The height of the wall ranged from 2.5 to 3.3 m. The longitudinal slope of the wall was up to 12°, and the transverse slope was up to 7°. A powered roof support was used in the wall, the working range of which was from 2.4 to 4.4 m.

**Figure 14:**
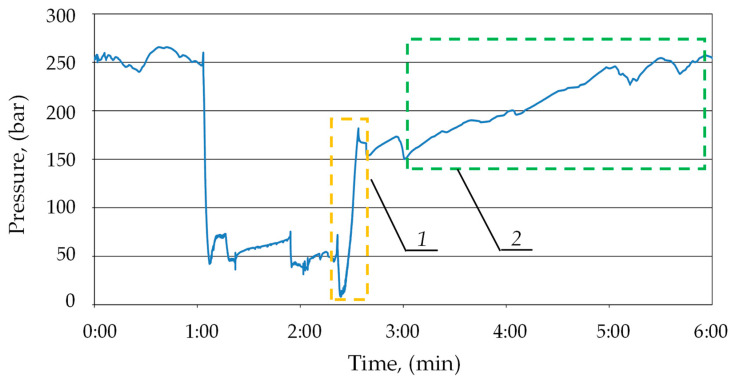
The course of pressure changes in the sub-piston space of a hydraulic stand with internal leakage (blue line) during the expansion operation: 1—expansion of the prop by the operator; and 2—automatic pressure charging.

**Figure 15:**
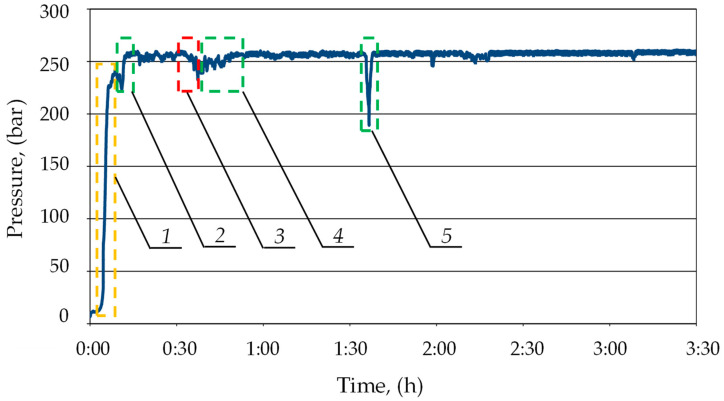
The course of pressure changes in the sub-piston space of a hydraulic prop with internal leakage (blue line) during its operation obtained in real tests: 1—expansion of the prop by the operator; 2—automatic pressure charging, 3—pressure loss in the space under the piston of the prop; and 4 and 5—automatic pressure charging.

For the study, we selected a prop with an internal leak. The standard valve block was replaced in this prop by a prototype double block with automatic pressure charging. In addition, the prop’s hydraulic system was equipped with wireless DOH-DROPS pressure sensors (Center of Hydraulics DOH Ltd., Bytom, Poland). These sensors continuously measured the pressure. The sampling rate was 0.01 s (100 measurements per second). The range of sensors’ operation was up to 60 MPa. The measurements allowed for generating time–pressure curves. Testing under real conditions allowed us to assess the usefulness of the block to ensure the correct operation of the powered roof support. Sample test results are presented in Figure 14 and Figure 15.

The research conducted in real conditions allowed us to evaluate three functions of the prototype block. The first function is expanding the prop to the initial load-carrying capacity value. The second function is to automatically charge the pressure in the prop’s sub-piston space if it drops. In addition, choosing a prop with an internal leak allowed us to evaluate the third function of the block—minimizing the effects of internal leaks. The results showed that the prototype block meets all the pre-supposed requirements.

Figure 14 presents the process of prop expansion. As one can see on the graph, the operator expanded the prop to a value of approx. 19 MPa (area 1). Then, after the end of the operator’s work, the block activated the charging. As a result, the pressure under the piston of the prop was increased to about 25 MPa (area 2). Thanks to this, the prop reached the required value of the initial load-carrying capacity, which confirms the correctness of the pre-supposed guidelines (the first function of the block).

Figure 15 shows the pressure measurement over 3.5 h. As can be seen, after installing the prototype block in the hydraulic system of the leaking prop, the pressure in the sub-piston space was maintained at the required level of min 25 MPa. The double block protected the prop’s over-piston and sub-piston space from liquid loss and pressure drops. This minimized the effects of internal leaks. On the chart, (see Figure 14), pressure drops in the sub-piston space of the prop may be observed (area 3). The drops might have resulted from the local conditions of cooperation between the powered roof support and the rock mass, as well as the pressure of rock layers. As you can see on the chart, after each pressure drop, the block automatically started charging (areas 4 and 5). This allowed the pressure in the sub-piston space to be replenished to the required value. This confirmed the correctness of the charging function. In area (1), the expansion of the section by the operator can be observed. The section was expanded to the load-carrying capacity of approx. 24 MPa (area 1). Therefore, the section did not reach the required load-carrying capacity. Therefore, the block automatically charged the pressure to a value of approx. 26 MPa (area 2).

**Figure 16:**
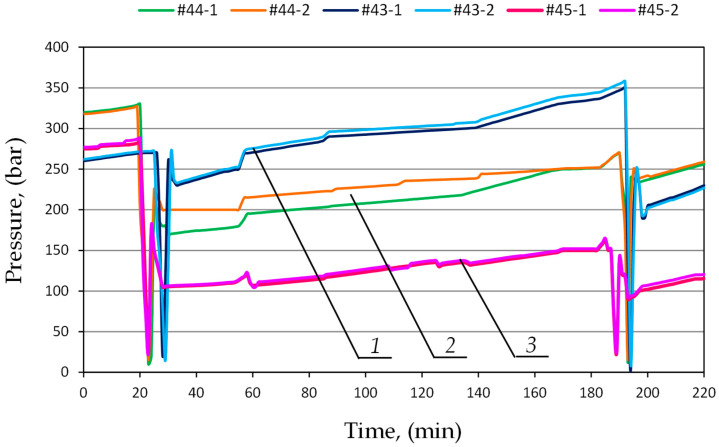
The course of pressure changes in the sub-piston space of the hydraulic prop, using a standard block for three adjacent sections, where 1—section No. 43; 2—section No. 44; and 3—section No. 45.

**Figure 17:**
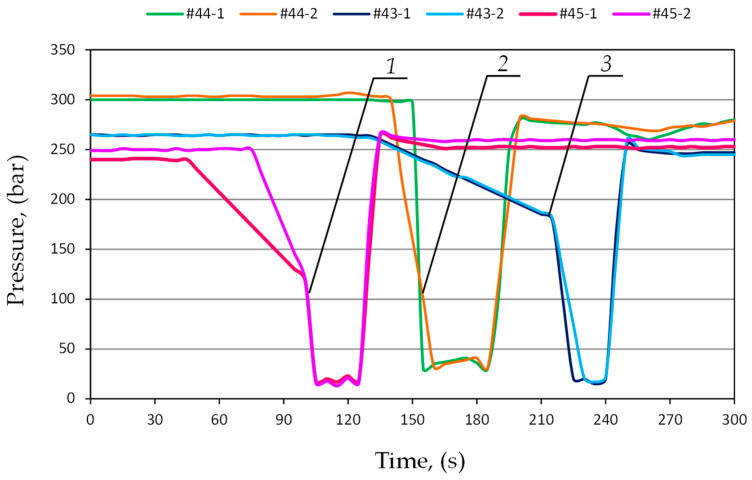
The course of pressure changes in the sub-piston space of the hydraulic stand using a prototype double block with automatic charging, where 1—section No. 45; 2—section No. 44; and 3—section No. 43.

The prototype double block with automatic pressure charging was compared with a standard valve block. For this purpose, further studies were carried out in the mining wall. The powered roof support is equipped with a wireless pressure monitoring system. The system made it possible to continuously measure the pressure in the prop’s sub-piston space. It allowed for constant monitoring of the powered roof support’s operation. The study included three adjacent sections of the powered roof support, numbered 43, 44 and 45. In the first stage of testing, the sections were equipped with a standard valve block. In the second stage, the traditional blocks were replaced with the prototype double blocks with automatic pressure charging. The research results made it possible to compare the performance characteristics of hydraulic props using both blocks. Examples of pressures for both test stages are shown in Figure 16 and Figure 17.

Figure 16 shows the flow of pressure changes in the prop’s sub-piston space for sections 43, 44 and 45, in which a standard block was used. The graph shows clear differences in pressure between the expanded sections. The difference between adjacent sections is 10 MPa. Not all sections reached the required load-carrying capacity. The pressure differences are repeated in the next operation cycle of the powered roof support.

In the second stage, double blocks with automatic charging were installed in the hydraulic system of the tested sections. Examples of pressure changes for sections 43, 44 and 45 are shown in Figure 17. The analysis of the research results showed that the work of the powered roof support is much more uniform. All sections operate at similar pressure values. Moreover, all sections reached the required load-carrying capacity. Minor pressure differences between successive sections may be due to the local conditions of cooperation between the powered roof support and the rock mass.

## 4. Discussion

**Figure 18:**
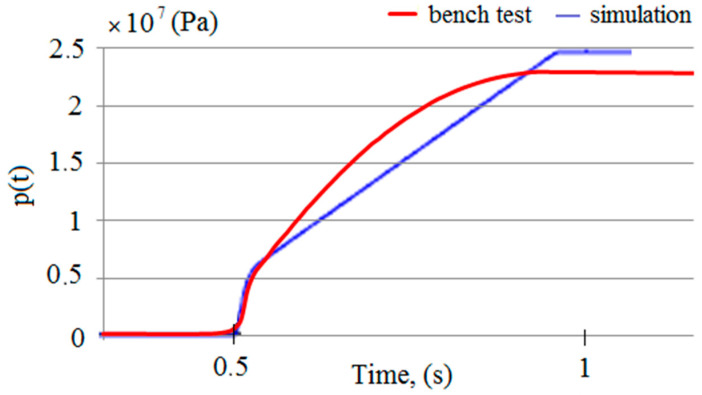
A summary of the results of simulation and bench tests for the operation of expanding the hydraulic prop in order to verify the adopted mathematical model.

**Figure 19:**
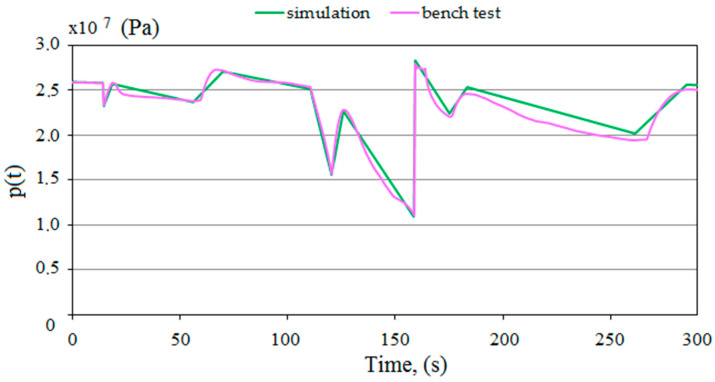
Summary of the results of simulation and bench tests for the pressure charging function in the hydraulic prop.

We had to verify the model to be sure of its correctness. For this purpose, the results of the model tests were compared with the results of the bench tests. In Figure 18, the obtained pressure changes in the sub-piston space during its expansion are summarized. The graphs from the model and bench tests are of the same nature. The lower pressure obtained in the bench tests is solely due to the power of the conveyor pump used for the bench testing. Figure 19 summarizes the pressure-change processes in the prop’s sub-piston space for the proposed charging function. There is a significant convergence of results between the model and bench tests. For model studies, the waveforms are linear, which is not observed for bench tests. This is due to the fact that bench tests are not carried out on an ideal system. Nonlinearities are affected by characteristics of the conveyor pump, possible contamination of liquids or lack of perfect sealing. These random variables are not included in the model because they do not significantly impact the nature of the charging function. The presented sets (see Figure 18 and Figure 19) show that a significant convergence was obtained in the model and bench tests. Thus, the bench test results confirmed the adopted model’s correctness.

**Figure 20:**
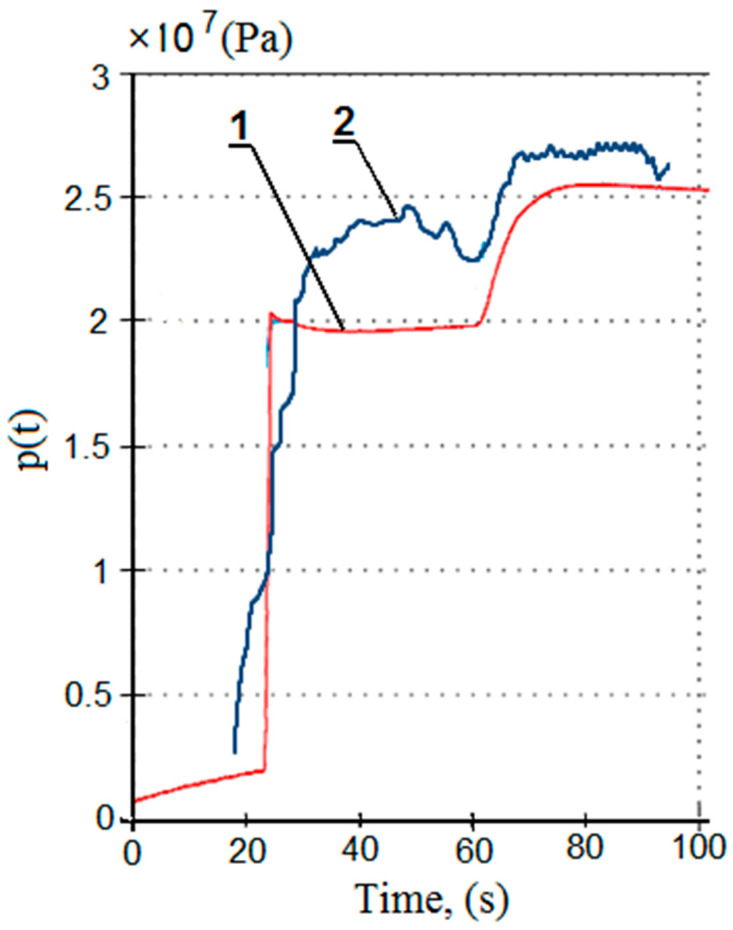
Summary of the results of bench tests (1) and tests in real conditions (2) for the pressure charging function.

Figure 20 presents the results of the bench and in-service tests for the proposed charging function. The pressure-flow changes obtained in the bench test are much more stable compared to the ones obtained in tests under real conditions. The results of the in-service tests are nonlinear; the graph shows continuous pulsation and pressure fluctuations. In real conditions, additional external forces acted on the tested prop—those derived from the load of the rock mass, the impact of the adjacent sections and the impact of the section’s other prop. For the bench tests, no external force acted on the prop. However, the charging process and the accompanying pressure changes in the sub-piston space were similar in both cases. The differences in the pressure values result from the irregular operation of the pumps and pressure fluctuations in the main supply line.

The current development of powered support includes adapting it to changing mining and geological conditions [35,36], including the exploitation of increasingly difficult seams [36]. Due to the increasing scale of the risk of rock bursts, many studies are focused on the dynamic loading of the powered roof support—mainly impact [45,46]. The authors of the article focused solely on ensuring the correct pressure in the hydraulic props, thus further ensuring the required load-carrying capacity force. The research did not take into account the influence of mining and geological conditions. The load on the rock mass was omitted. The solution developed and presented in the article is intended to be universal and can be used both in rock burst hazard conditions and in longwalls where there is no risk of rock mass tremors. However, all of these global studies have one common goal—the optimization of powered roof support to improve occupational safety.

## 5. Conclusions

Exploiting increasingly lower coal beds generates more load on the part of the rock mass. The powered roof support must then be adapted to changing working conditions. Reliable operation of the powered roof support is necessary to ensure safety in the mining wall. One of the problems in operating a powered roof support is obtaining the required value of the load-carrying capacity. For this reason, the authors of the article proposed using a prototype double block with automatic pressure charging for the powered roof support hydraulic system. The research was planned and carried out for this specific issue. The research included computer simulations, bench tests and tests in real conditions. The results of the conducted studies and their analysis allowed us to draw the following conclusions:

(1) At the stage of creating the new prototype, mathematical modeling and simulation studies allowed us to determine its performance characteristics. The mathematical model and the simulation studies made it possible to quickly verify the correctness of the assumptions. Studies have shown that the proposed pressure charging function meets the accepted assumptions.
(2) During the bench tests, no problems were found in implementing the charging function. Using a double block with automatic pressure charging ensured that the required pressure value was maintained in the sub-piston space of the prop. Studies have shown that the double block should achieve the required operating parameters under real conditions.
(3) The results of the tests under real conditions confirmed the correctness of the proposed changes for introducing a double block with charging into the hydraulic system of the powered roof support. The prototype block performed its functions correctly under real conditions, minimizing the effects of internal leaks and ensuring that the required load-carrying capacity was reached and maintained.
(4) Comparing the work of the standard block with the prototype block, the authors’ solution ensures uniform work of the powered roof support. Powered roof support sections operate at similar pressures. After using the prototype block, the sections achieved the required initial and working load-carrying capacity.

The solution proposed by the authors can be classified as technical support in coal mining. The solution improves the reliability of the powered roof support. Thus, it provides improved safety in the mining wall. Currently, preparatory research is being carried out to introduce the target solution. This will be the next research stage from which statistical results will be developed to determine the distribution of the load-carrying capacity of the powered roof support along the length of the wall. Then, the prototype block can be used to develop an automatic prop expansion system. Such a solution would reduce the time of the expansion, which is essential regarding the amount of daily extraction.

## Data Availability

Data are contained within the article.
